# Involvement of Transient Receptor Potential Cation Channel Member A1 activation in the irritation and pain response elicited by skin-lightening reagent hydroquinone

**DOI:** 10.1038/s41598-017-07651-5

**Published:** 2017-08-08

**Authors:** Yan Tai, Chuan Wang, Zhihua Wang, Yi Liang, Junying Du, Dongwei He, Xiaoyan Fan, Sven-Eric Jordt, Boyi Liu

**Affiliations:** 1 Laboratory and Equipment Administration, Zhejiang Chinese Medical University, Hangzhou, 310053 China; 2 Department of Pharmacology, Hebei Medical University, Shijiazhuang, 050017 China; 3 Department of Neurobiology and Acupuncture Research, The Third Clinical Medical College, Zhejiang Chinese Medical University, Hangzhou, 310053 China; 4 Department of Clinical Bio-Cell, 4th Hospital, Hebei Medical University, Shijiazhuang, 050000 China; 5 Department of Oncology, Hebei General Hospital, Shijiazhuang, 050000 China; 6 Department of Anesthesiology, Duke University School of Medicine, Durham, North Carolina 27710 United States of America

## Abstract

Hydroquinone (HQ) is one of the most frequently used and effective skin-lightening products to treat skin hyperpigmentation disorders, including postinflammatory hyperpigmentation, melasma and solar lentigines. HQ is also widely used in cosmetic products for skin whitening. However, HQ treatment can evoke substantial skin irritation, a side effect that remains poorly understood. Here we demonstrate that HQ is an activator of the peripheral irritant receptor transient receptor potential (TRP) cation channel member A1 (TRPA1). HQ failed to activate TRPV1, TRPV4 or TRPM8. HQ-induced TRPA1 activation was dependent on essential redox-sensitive cysteine and lysine residues within N-terminus of channel protein. HQ elicited Ca^2+^ influx in a subpopulation of mouse sensory neurons sensitive to the TRPA1 agonist, mustard oil. HQ-induced neuronal responses were significantly reduced by TRPA1 inhibitors, and reduced in neurons isolated from Trpa1-deficient mice. In mice, intraplantar injection of HQ at clinically relevant concentrations elicited both acute pain and persistent mechanical hyperalgesia which were almost completely abolished by TRPA1 inhibitors. These findings identify TRPA1 as a molecular target for HQ and provide insights into the mechanism of HQ-induced skin irritation. These findings also suggest that selective TRPA1 antagonists may be useful to counteract HQ-induced skin irritation.

## Introduction

Although common and mostly benign, skin hyperpigmentation disorders, including postinflammatory hyperpigmentation (PIH), melasma and solar lentigines, oftentimes present significant cosmetic or psychological challenges to the patient^1, 2^. Hydroquinone (HQ) is the active ingredient in the most frequently used and effective skin-lightening products for the treatment of skin hyperpigmentation disorders on the market^3–5^. HQ is also widely used in North America, Europe, Asia, and Africa countries for cosmetic skin whitening purpose^6^. It has been estimated that 10–15 million tubes of skin-lightening formulations containing HQ are sold worldwide annually^7^. HQ exerts its therapeutic effects via multiple mechanisms, including: 1) inhibition of the enzymatic oxidation of tyrosine and phenol oxidases; 2) covalent binding to histidine and interaction with the active site of tyrosinase; 3) inhibition of RNA and DNA synthesis^7^. These effects result in selective damage of melanocytes and suppression of melanin pigment production^7^. Although some concerns of possible carcinogenicity and disfiguring ochronosis have been raised in association with long term HQ usage, analysis has remained inconclusive^3^. The US FDA has classified formulations with 1.5–2% HQ content as over the counter (OTC) treatments, whereas treatments with >4% HQ are only available by prescription^4, 5^. HQ remains the gold standard treatment for PIH, melasma and solar lentigines^3, 4^.

Despite the beneficial effects on skin hyperpigmentation, HQ can cause substantial skin irritating side effects among patients^8–10^. Topical HQ often causes local irritation, including burning, pruritus and erythema^3^. Oftentimes formulations contain topical steroids to suppress HQ-elicited irritation^2, 3^. However, long-term usage of topical steroids can cause skin side effects as well, including skin atrophy^11^. Animal studies have confirmed that topical HQ caused obvious skin irritation^12^. The molecular and cellular mechanisms mediating HQ-induced skin irritation remain largely unknown. Understanding the mechanisms underlying HQ-induced irritation may facilitate the development of effective methods to counteract the irritating side effects of HQ treatment and improve the patients’ compliance.

TRPA1 is a non-selective cation ion channel exclusively expressed in nociceptive sensory neurons where it acts as molecular sensors for painful, irritating and pruritic stimuli^13^. Mammalian TRPA1 can be robustly activated by a wide variety of endogenous/exogenous substances that elicit pain, itch and irritation^14–17^. TRPA1 contributes to the perception of noxious stimuli and plays an important role in sensory transduction. Activation of TRPA1 can further produce neurogenic inflammation, which is elicited by neuropeptides released from sensory nerve endings, including substance P (SP) and calcitonin gene-related peptide (CGRP)^13, 18, 19^. Pharmacological blockage or genetic ablation of TRPA1 can reduce the acute response caused by many painful or irritating substances^15, 16, 20, 21^.

In the present study we examined the effects of HQ on TRPA1 heterologously expressed in HEK 293 cells *in vitro* by means of Fura-2 based ratiometric Ca^2+^ imaging and whole-cell patch clamp recording. We further examined the effects of HQ on cultured sensory neurons derived from wild-type and TRPA1-deficient mice. Lastly, we investigated whether TRPA1 contributes to HQ-induced irritation and nocifensive responses in mice *in vivo*. Our study demonstrates that HQ is a potent agonist of TRPA1 and produces pain or irritation mainly through activation of TRPA1. Local pharmacological block of TRPA1 may provide an efficient way to reduce the skin irritation caused by HQ, thereby improving patients’ compliance when treated with HQ-containing skin therapeutics.

## Results

### HQ dose dependently and selectively activates human TRPA1 ion channels heterologously expressed in HEK 293 cells

**Figure 1:**
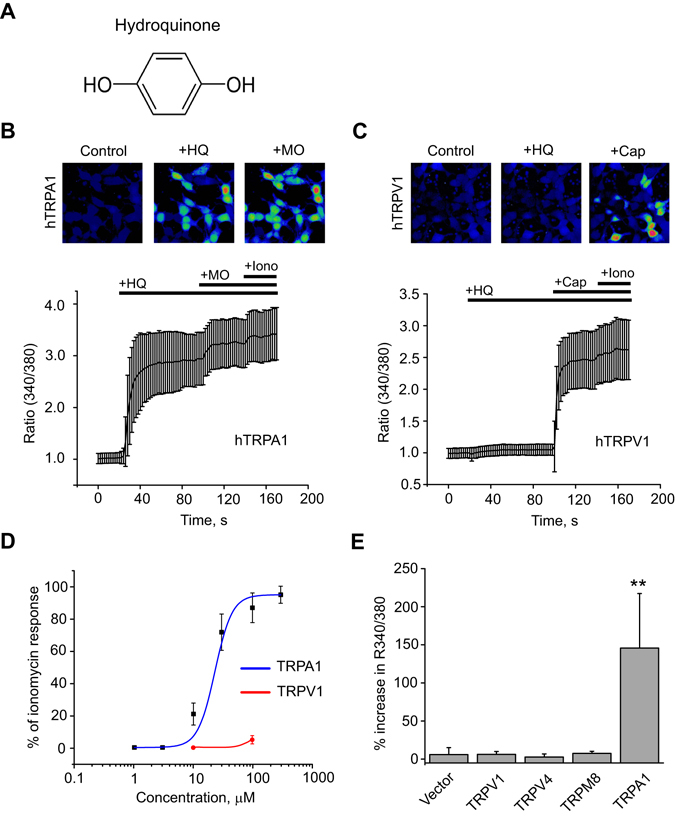
HQ selectively and dose-dependently actives TRPA1 channel heterologously expressed in HEK 293 cells. (**A**) Molecular structure of HQ. (**B**) Top: Representative pseudo color images from Fura-2-loaded HEK 293 cells expressing human TRPA1, displaying intracellular Ca^2+^ resonses. Cells are shown in resting state (left), followed by response to HQ (HQ, 100 µM, middle) and to mustard oil (MO, 70 µM, right), used as positive control to stimulate TRPA1. Bottom: Average Ca^2+^ responses plotted against stimulus. As final stimulus ionomycin (1.5 µM) was used to stimulate all live cells. (**C**) Examination of responsiveness of human TRPV1 to HQ (HQ, 100 µM) and capsaicin (Cap, 300 nM), as positive control. (**D**) Dose-response analysis showing the effects of different concentrations of HQ on TRPA1 and TRPV1 expressed in HEK 293 cells. The response to HQ was normalized to that to ionomycin (1.5 µM). (**E**) Effects of HQ (30 µM) on hTRPA1 compared to effects on human TRPV1, TRPV4, TRPM8 and empty vector (pcDNA3.1) in Ca^2+^ imaging tests. n = 20–40 cells/group. **p < 0.01 *vs*. vector group.

HQ is a phenolic compound chemically known as 1, 4-dihydroxybenzene (Fig. 1A). We aimed to explore whether HQ could act as an activator of the irritation/pain-sensing TRPA1 or TRPV1 ion channels *in vitro*. Live cell Ca^2+^ imaging was used to investigate the effects of HQ on HEK 293 cells expressing either human TRPA1 (hTRPA1) or human TRPV1 (hTRPV1). Fura-2AM was used as Ca^2+^ indicator. Ca^2+^ imaging demonstrated that HQ (100 µM) caused significant influx of Ca^2+^ in HEK 293 cells expressing hTRPA1 (Fig. 1B). Subsequent application of mustard oil (MO, 70 µM), a well-established TRPA1 agonist, caused further influx of Ca^2+^ in HQ-responsive cells, demonstrating that HQ exclusively acted on TRPA1-expressing cells (Fig. 1B). In contrast, HQ had no effect on HEK 293 cells expressing hTRPV1, whereas the TRPV1 specific agonist capsaicin robustly activated TRPV1-expressing cells (Fig. 1C).

We continued to explore whether HQ activated hTRPA1 in a dose-dependent manner. TRPA1-mediated Ca^2+^ influx was increased at higher HQ concentrations, while HQ had no effect on TRPV1 within the concentration range (10–100 µM) being tested (Fig. 1D). The EC_50_ of HQ-induced hTRPA1 activation deduced from the curve was determined to be 17.1 µM (Fig. 1D). We proceeded to study the selectivity of HQ by comparing its effects on TRPV4 and TRPM8, two other TRP ion channels expressed in sensory nerves or skin cells, using empty vector (pCDNA3.1) as control. Result showed that HQ only activated TRPA1, but none of the other TRP channels or the empty vector-transfected cells (Fig. 1E).

**Figure 2:**
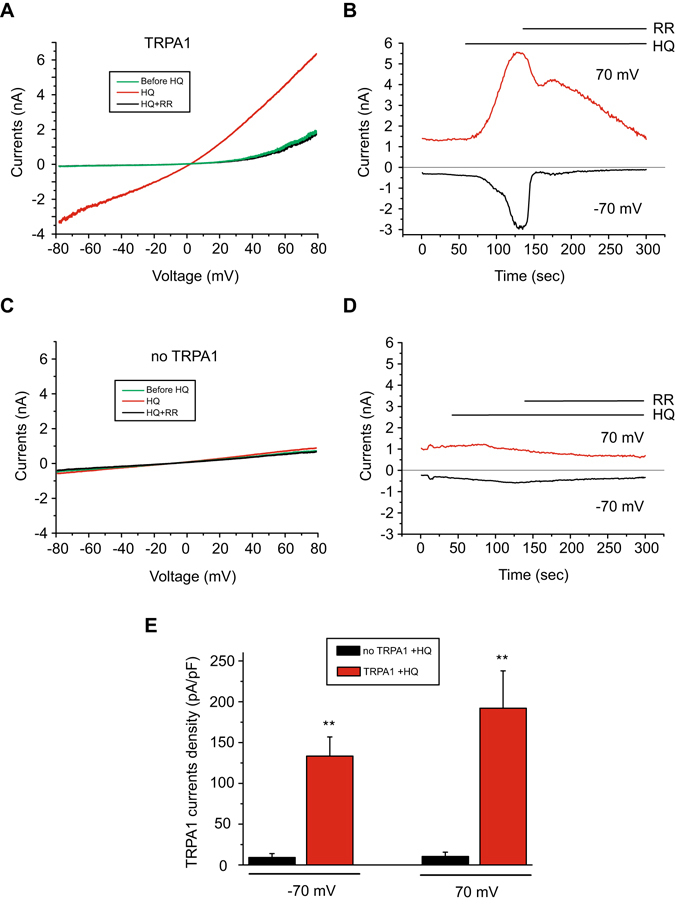
HQ evokes TRPA1 channel currents in HEK 293 cells expressing TRPA1. (**A**) Current-voltage (I-V) curve recorded from a representative HEK 293 cell expressing human TRPA1 before HQ application (green), during HQ application (red) and inhibition by ruthenium red (10 µM, black) in the patch clamp whole-cell configuration. Currents were measured by applying a voltage ramp ranging from −80 to + 80 mV. (**B**) Time courses of the inward (measured at −70 mV, black) and outward current (measured at +70 mV, red) recorded in (**A**). (**C**) I-V curve recorded under the same configuration as in (**A**), except that the cell was transfected with empty vector. (**D**) The time course of the inward (measured at −70 mV) and outward current (measured at +70 mV) as recorded in (**C**). (**E**) Summary of the inward and outward current density recorded at −70 and +70 mV, respectively, in HEK 293 cells expressing hTRPA1 or empty vector. **p < 0.01 *vs*. vector group.

To confirm that HQ activated TRPA1, we examined the effects of HQ on TRPA1-expressing HEK 293 cells by whole-cell patch-clamp electrophysiological recordings. To avoid TRPA1 channel inactivation, channel currents were recorded using Ca^2+^-free extracellular solution^22^. As shown in Fig. 2A, HQ (10 µM) induced robust activation of the hTRPA1-mediated cellular currents with strong inward and outward currents measured at −70 and +70 mV, respectively (Fig. 2B). The inward and outward currents induced by HQ were strongly inhibited by ruthenium red (RR, 10 µM), a nonspecific TRP channel blocker (Fig. 2A and B). As a negative control, HQ had no effect on HEK 293 cells transfected with empty vector (pCDNA3.1) alone (Fig. 2C and D). On average, the amplitudes of inward and outward currents induced by HQ (10 µM) in HEK 293 cells expressing hTRPA1 reached 133 and 192 pA/pF, respectively, whereas HQ induced no detectable currents in cells expressing the vector alone (Fig. 2E). Taken together, the Ca^2+^ imaging and patch clamp recording data demonstrate that HQ specifically and dose-dependently activates hTRPA1 heterologously expressed in HEK 293 cells.

### HQ activates TRPA1 through covalent modification of reactive residues located in the N-terminus of the channel protein

**Figure 3:**
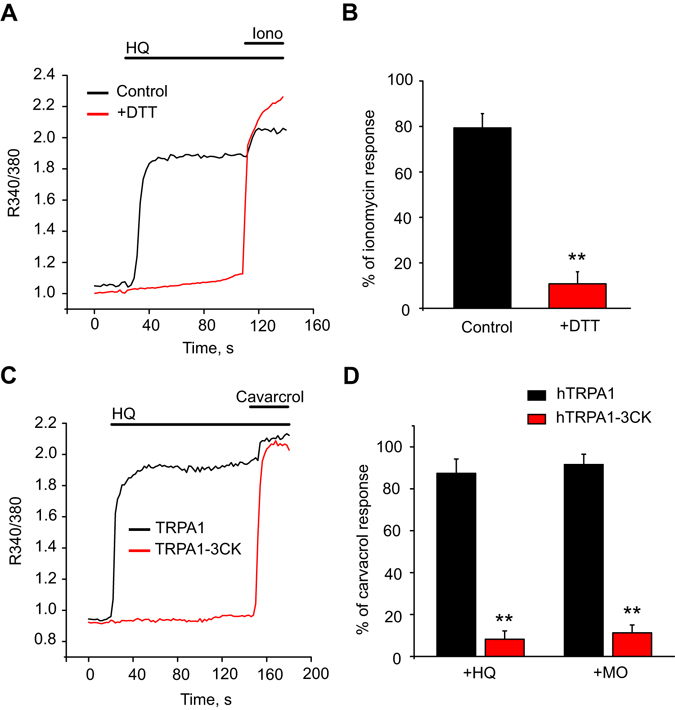
Covalent modification sites of TRPA1 are essential for the effects of HQ. (**A**) Representative Ca^2+^ imaging traces showing the effects of reducing reagent DTT on HQ-induced hTRPA1 activation in HEK 293 cells. Cells were pretreated with dithiothreitol (DTT, 3 mM) for 3 min before recording and then recorded under the continued presence of DTT. Cells were challenged with HQ (30 µM) and subsequently with ionomycin (Iono, 1.5 µM). (**B**) Summary of the effects of DTT on HQ-induced Ca^2+^ responses in HEK 293 cells. n > 40 cells/group. **p < 0.01 *vs*. Control group. (**C**) Representative Ca^2+^ imaging traces showing responses of hTRPA1 and hTRPA1 mutant (3CK mutant)-expressing HEK293 cells following application of HQ (30 µM) and carvacrol (300 μM). (**D**) Averaged Ca^2+^ responses induced by HQ and mustard oil (MO, 70 µM) in hTRPA1 and hTRPA1–3CK. The response was normalized to that of a saturating dose of carvacrol (300 μM). n > 40 cells/group. **p < 0.01 *vs*. hTRPA1 group.

It is well established that TRPA1 channel can be activated by a variety of reactive chemicals through covalent modification of cysteine and lysine residues in the N-terminus of the channel protein. Therefore, we aimed to ask whether HQ activated TRPA1 via similar mechanisms. We first examined the effects of dithiothreitol (DTT), a reducing reagent, on HQ-induced TRPA1 activation in HEK 293 cells by Ca^2+^ imaging. As shown in Fig. 3A, HQ (30 µM) induced robust activation of hTRPA1. Pretreatment of cells with DTT (3 mM) almost totally abolished HQ-induced hTRPA1 activation (Fig. 3A and B). Next, we examined whether cysteine and lysine residues in the N-terminus of the channel protein were involved in HQ-induced TRPA1 activation. HEK 293 cells were transfected with a mutant TRPA1 channel in which critical reactive sites (C619, C639, C663, and K708, termed as TRPA1–3CK) were replaced by inert residues. These mutations have been shown to significantly reduce TRPA1 activation by many reactive chemicals^15–17^. As shown in Fig. 3C and D, TRPA1-3CK transfected cells showed almost no response to HQ, compared with wild type hTRPA1-transfected cells. For comparison, the effect of mustard oil (70 µM), a typical reactive TRPA1 agonist, was significantly reduced in TRPA1-3CK-transfected cells, in confirmation with previous studies (Fig. 3D). TRPA1-3CK channel remained functional since carvacrol (300 µM), a nonreactive agonist that activated TRPA1 via non-covalent mechanism, robustly activated the channel (Fig. 3C)^23^. Therefore, the above results demonstrate that HQ activates TRPA1 via covalent modification of cysteine and lysine residues in the N-terminus of the channel protein.

### TRPA1 expressed in mouse primary sensory neurons is a molecular target of HQ

**Figure 4:**
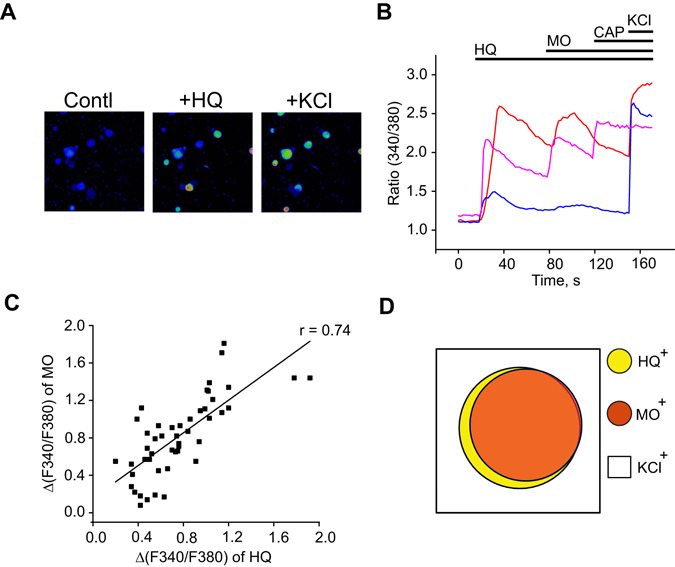
HQ triggers Ca^2+^ responses in mouse sensory neurons. (**A**) Representative pseudo-color images of the effect of HQ (30 µM) on intracellular Ca^2+^ of cultured mouse DRG neurons. (**B**) Representative Ca^2+^ imaging traces showing the effect of HQ on cultured mouse DRG neurons. The neurons were first challenged with HQ (30 µM) and subsequently with mustard oil (MO, 100 µM), capsaicin (CAP, 200 nM) and KCl (40 mM). (**C**) The correlation of the magnitude of HQ- and MO-induced intracellular Ca^2+^ signal in mouse DRG neurons (r = 0.74 and P < 0.000001, Pearson correlation, n = 49 neurons). (**D**) Venn diagram showing the overlap of neuronal populations responding to HQ, MO and Cap in mouse DRG neurons. Size and overlap of populations are indicated by circles drawn to the actual scale. 67, 57 and 156 neurons are included in HQ^+^, MO^+^ and KCl^+^ groups, respectively.

We proceeded to explore whether HQ stimulated primary sensory neurons through activation of TRPA1 *in vitro*. Cultured mouse dorsal root ganglion (DRG) neurons were loaded with Fura-2 for ratiometric Ca^2+^ imaging. We observed that HQ (30 µM) induced robust Ca^2+^ signals in a subset of mouse DRG neurons (Fig. 4A and B). A majority of the HQ-responsive neurons showed further Ca^2+^ responses upon subsequent application of a saturating dose of TRPA1 agonist MO (100 µM) (Fig. 4B). To further investigate if HQ-induced Ca^2+^ responses were predominately distributed in neurons expressing TRPA1, we evaluated the correlation of MO-induced Ca^2+^ response with HQ-induced Ca^2+^ response in mouse DRG neurons. Pearson correlation analysis revealed that a significant positive correlation existed between the magnitude of Ca^2+^ responses induced by HQ and MO (r = 0.74 and p < 0.000001, Fig. 4C). Venn diagram analysis revealed a >80% overlap of the HQ-responsive neuronal population (HQ^+^) with the MO-responsive neuronal population (MO^+^).

**Figure 5:**
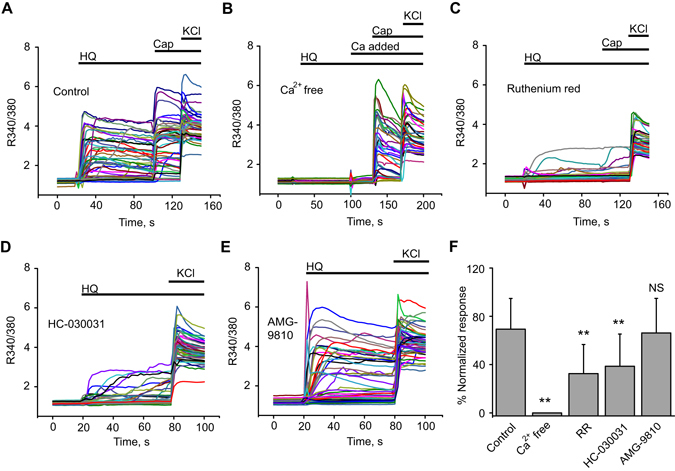
Pharmacological analysis of HQ-induced Ca^2+^ responses in mouse sensory neurons. (**A**–**E**) Overlaid Ca^2+^ imaging traces induced by HQ in control condition (**A**) and in the presence of Ca^2+^ free extracellular solution (**B**), ruthenium red (10 µM) (**C**), HC-030031 (100 µM) (**D**) and AMG-9810 (6 µM) (**E**) in mouse DRG neurons. Neurons were treated with HQ (30 µM), and subsequently with capsaicin (200 nM) and KCl (40 mM) as shown. 2 mM Ca^2+^ was re-added as shown in (**B**). (**F**) Summary of the pharmacological studies as shown in (**A–E**). All HQ-induced Ca^2+^ responses were normalized to that of the KCl response and shown as % normalized response. n > 40 cells/group. **p < 0.01 *vs*. control group. NS: no significance (p > 0.05).

We next used additional pharmacological tools to examine whether TRPA1 was involved in HQ-induced Ca^2+^ responses in DRG neurons. HQ-induced Ca^2+^ responses were completely abolished when experiments were performed in Ca^2+^-free extracellular solution (Fig. 5A,B). The broad spectrum TRP channel blocker ruthenium red (RR, 10 µM) significantly reduced the magnitude of HQ-induced Ca^2+^ response (Fig. 5C). Furthermore, the selective TRPA1 inhibitor HC-030031 (100 µM) significantly reduced the magnitude of the HQ-induced Ca^2+^ response to a similar extent as RR (Fig. 5D). Minor residual Ca^2+^ responses remained during inhibitor treatments with RR or HC-030031 (Fig. 5C and D). The TRPV1-specific inhibitor, AMG-9810, at the effective dosage that blocked TRPV1 *in vitro* (6 µM) had no effect on HQ-induced Ca^2+^ responses (Fig. 5E)^24, 25^. The magnitudes of HQ-induced Ca^2+^ responses during different pharmacological treatments are summarized in Fig. 5F.

**Figure 6:**
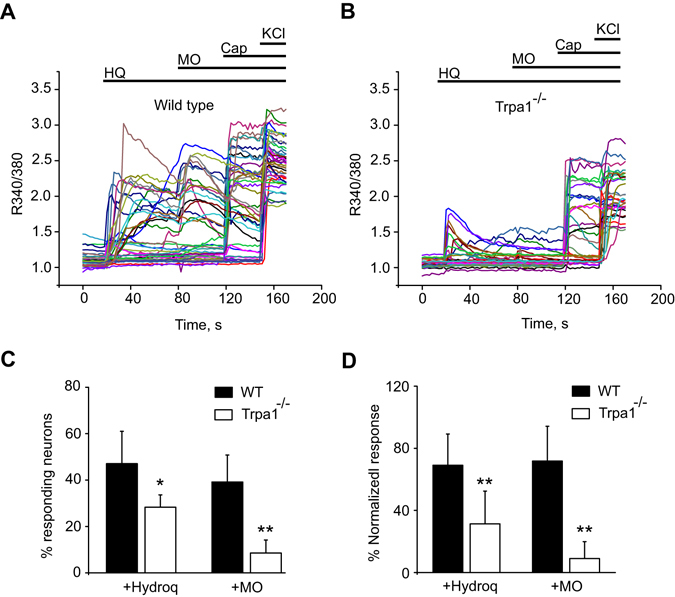
HQ activates TRPA1 in mouse sensory neurons. (**A**) Representative pseudo-color images of the effect of HQ (30 µM) on intracellular Ca^2+^ of cultured DRG neurons isolated from TRPA1 deficient mice. (**B**) Representative Ca^2+^ imaging traces showing HQ responses in cultured DRG neurons isolated from Trpa1^−/−^ mice. Drug dosages and application procedures are the same as in (**A**). (**C**) Averaged percentages of neurons from wild type(WT) or Trpa^−/−^ mice positively responding to HQ and MO. (D) Averaged percent increases of R_340/380_ of the Ca^2+^ responses to HQ and MO recorded from neurons isolated from WT or Trpa1^−/−^ mice. n = 5–6 tests/group, each group contains up to 30 neurons. *p < 0.05, **p < 0.01.

We further evaluated the contribution of TRPA1 in HQ-induced Ca^2+^ responses using TRPA1-deficient (Trpa1^−/−^) mice. DRG neurons were isolated and cultured from WT and Trpa1^−/−^ mice and subjected to Ca^2+^ imaging. We found that the percentage of HQ-responsive neurons and the amplitude of HQ-induced Ca^2+^ responses were both significantly reduced in neurons isolated from Trpa1^−/−^ mice compared with WT neurons (Fig. 6A–D). For control, the neurons cultured from Trpa1^−/−^ mice showed significantly reduced MO-induced Ca^2+^ response compared with WT, while the effect of capsaicin (acting via TRPV1) remained unaltered (Fig. 6A–D). Overall, the above results demonstrate that TRPA1 is critically involved in the Ca^2+^ responses induced by HQ in DRG neurons *in vitro*.

### HQ induces both acute and persistent nocifensive behavior *in vivo* through TRPA1-dependent mechanism

**Figure 7:**
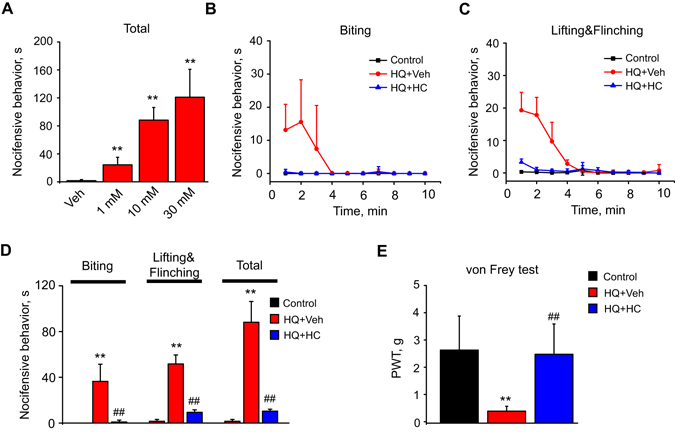
HQ induces acute nocifensive behavior and long-lasting mechanical hyperalgesia through a TRPA1-dependent mechanism in mice. (**A**) HQ dose-dependently elicited nocifensive behavior in mice when injected into the hind paw. Vehicle (PBS) or 1 mM, 10 mM, 30 mM HQ in 20 µl volume was administered to the hind paw through intraplantar injection. Total nocifensive responses (the time mouse spent on paw biting, lifting and flinching) were summarized. n = 5–8 mice/group. (**B,C**) Time course of mouse biting (**B**), lifting & flinching (**C**) behavior after intraplantar injection of vehicle (Control), HQ (HQ, 10 mM) + vehicle or HQ (HQ, 10 mM) + HC-030031 (HC, 10 µg). (**D**) Averaged effect of HC-030031 on HQ-induced nocifensive behavior, including biting, lifting&flinching and total. (**E**) Paw withdraw threshold (PWT) of mice measured by von Frey hair testing. Tests were carried out 1.5 hour after vehicle, HQ + Veh or HQ + HC-030031 injection into the hind paw. n = 7–8 mice/group. **p < 0.01 *vs*. vehicle or control group, ^##^p < 0.01 *vs*. HQ group.

Since HQ can stimulate mouse primary sensory neurons via TRPA1 activation *in vitro*, we carried out animal behavioral assays to examine whether HQ could actually elicit irritation or pain-related behavior in mice *in vivo* and if so, whether it was related to TRPA1 activation. Intraplantar injection of HQ (3 to 30 mM, in 20 µl volume) dose-dependently elicited biting, lifting and flinching behavior in mice, all signs of acute pain (Fig. 7A). In contrast, vehicle (PBS)-injected mice showed minimal responses (Fig. 7A). These acute nocifensive behaviors occurred immediately after HQ injection and gradually declined within 5 min (Fig. 7B and C). The total time the mice spent biting, lifting and flinching the injected paw after HQ or vehicle injection is summarized in Fig. 7A.

We next examined whether administration of the TRPA1 specific inhibitor, HC-030031, could reduce HQ-induced acute nocifensive behavior in mice. Co-injection of HC-030031 (10 µg) dramatically reduced HQ (10 mM, in 20 µl volume)-induced biting, lifting and flinching behavior. The effect of HC-030031 on HQ-induced acute nocifensive behavior is summarized in Fig. 7D. We proceeded using von Frey hair-analysis to investigate whether HQ could produce persistent pain through activation of TRPA1 *in vivo*. The mice still exhibited obvious signs of mechanical hyperalgesia measured 1.5 h after HQ injection (10 mM, Fig. 7E). The persistent mechanical hyperalgesia induced by HQ was strongly reduced by the TRPA1 antagonist, HC-030031 (Fig. 7E). Therefore, the above results demonstrated that HQ could cause both acute pain and persistent mechanical hyperalgesia via activation of TRPA1.

## Discussion

In the present study, we identified hTRPA1 as a novel molecular target for the skin-lightening reagent HQ both *in vitro* and *in vivo*. HQ elicited robust pain or irritation responses in mice through a TRPA1-dependent mechanism. These conclusions are based on the following findings: First, HQ dose-dependently activated hTRPA1 heterologously expressed in HEK 293 cells. This effect on TRPA1 was specific since HQ exerted no obvious effect on other TRP channels, including TRPV1, TRPV4 or TRPM8. Second, HQ only activated a subpopulation of mouse DRG neurons, which correlated closely with mustard oil-responsive neurons. Third, HQ-induced Ca^2+^ responses in mouse DRG neurons were significantly attenuated by pharmacological blockage of TRPA1 or by genetic deletion of TRPA1. Lastly, *in vivo* animal behavioral tests showed that HQ induced both acute pain and persistent mechanical hyperalgesia in mice. These nocifensive behaviors were almost completely attenuated by locally treating animals with TRPA1 specific antagonist HC-030031.

HQ is usually applied in ointments or creams to the skin for hyperpigmentation disorder treatment. A previous study investigated the intradermal concentration of clinically used HQ (5.0%) after its permeation using rat skin^26^. In that study, it was reported that the intradermal concentrations at 2 h after application of the HQ ointments were 358 mM in stratum corneum and 51.7 mM in epidermis plus dermis tissue^26^. In the present study, we found that HQ can robustly activate TRPA1 currents in HEK 293 cells at concentrations as low as 10 μM and the estimated EC_50_ by Ca^2+^ imaging is 17.1 μM, which is far less than the concentration of HQ detected in the skin treated with a clinical ointment. Therefore, it can be expected that topically applied HQ ointment will lead to local HQ concentrations that by far exceed the threshold needed for TRPA1 activation. Our study did not test whether topical application of an HQ-containing ointment can cause irritation or pain response in the hind paw of mice, because mice usually lick, bite and scratch the application site to remove the ointment. Previous studies using black guinea pigs have demonstrated that topical HQ application can cause skin irritation. Furthermore, our *in vivo* studies demonstrated that intraplantar injection of HQ at a concentration range of 1 to 30 mM can readily produce both acute pain and persistent mechanical hyperalgesia in mice. This concentration range is much lower than the concentration of HQ found in stratum corneum and epidermis plus dermis tissue (358 and 51.7 mM, respectively) when 5% clinically used HQ ointment is applied on the skin^26^. Therefore, it is quite likely that topically applied HQ ointment in patients, containing much higher amounts of HQ, will activate TRPA1 and cause irritation and pain.

Although TRPA1 specific inhibitors or TRPA1 gene deficiency significantly reduced HQ-induced Ca^2+^ responses in mouse DRG neurons, residual Ca^2+^ responses remained under these conditions. This observation suggests that other mechanisms, in addition to TRPA1 activation, contribute to the HQ-induced Ca^2+^ responses in DRG neurons. The cellular HQ-induced Ca^2+^ response does not derive from intracellular Ca^2+^ release since it can be completely blocked by removing extracellular Ca^2+^. The broad spectrum TRP channel blocker ruthenium red could not completely block HQ-induced Ca^2+^ responses, suggesting that the residual response is not mediated by TRP channels. While we are currently unable to pinpoint the molecular identity of this residual activity, it may contribute to the painful and irritating effects of HQ by eliciting neuronal excitation and neurogenic release of pro-inflammatory neuropeptides. While not being the sole molecular target of HQ in sensory neurons, *in vivo* pharmacological blockage of TRPA1 resulted in an almost complete abolishment of HQ-induced acute irritation/pain response and persistent mechanical hyperalgesia in mice. Therefore, the present results suggest that TRPA1 acts as a major molecular target of HQ and plays a predominant role in mediating the irritation or pain response induced by HQ.

Previous studies identified TRPA1 immune reactivity in cells of the human skin, implying that TRPA1 may act as an irritant receptor in skin cells^27^. However, other studies, including ours, did not detect significant TRPA1 expression in neither skin cells nor cells of the immune system, while abundant expression is observed in sensory nerves^28–30^. To validate these findings we recently applied a systematic approach using real-time PCR and digital droplet PCR that failed to detect the presence of TRPA1 mRNA in skin samples from mice and humans. Expression of other TRP channels, such as TRPV4, was readily detectable^14^. While further detailed analysis of TRPA1 expression in skin or skin cells is needed, these data suggest that skin cells produce minimal amounts of TRPA1, and the majority of TRPA1 in the skin is localized to sensory nerve endings.

It is interesting to note that zinc, which also has effects on melanogenesis^31, 32^ and, similar to HQ, is used for treatment of skin hyperpigmentation, robustly activates TRPA1 and causes irritation as well^33^. While zinc likely affects melanocytes directly, it remains to be investigated whether TRPA1 activation is essential for the skin lightening effects of both zinc and HQ, potentially by triggering neuropeptide release that may affect melanogenesis. Recent studies observed that CGRP and Substance P, both neuropeptides known to be released from nerve endings upon TRPA1 stimulation, inhibit melanogenesis and induce melanocyte apoptosis^29, 34, 35^. The mechanisms of TRPA1 activation by these two chemicals are significantly different. We identified cysteine and lysine residues in the N-terminus of the channel protein (C619, C639, C663, and K708) as crucial residues for TRPA1 activation by HQ, whereas cysteine and histidine residues (C1021, H983 in the C-terminus and C641 in the N-terminus) are crucial for TRPA1 activation by zinc^33^. Therefore, HQ and zinc activates TRPA1 via different mechanisms.

HQ exerts its therapeutic effects mainly in melanocytes with active tyrosinase activity, such as epidermal hyperactivated melanocytes^36^. The oxidation products of hydroquinone are quinones and reactive oxygen species, which lead to an oxidative damage of membrane lipids and proteins of melanocytes, including tyrosinase, which is the main enzyme regulating melanin synthesis^37, 38^. In addition, HQ may interfere with skin pigmentation even through multiple mechanisms, including (i) the covalent binding to histidine or interaction with coppers at the active site of tyrosinase, (ii) the inhibition of DNA and RNA synthesis and (iii) the alteration of melanosome formation and melanization extent^7^.

However, due to the widespread use of HQ in prescription, OTC and cosmetic formulations, there is considerable concern about its potential dermatological and systemic side effects, especially during long term usage. Especially nephrotoxicity, carcinogenicity and disfiguring ochronosis may be associated with HQ use^7^. Therefore, HQ is banned in the European Union (EU) as an ingredient in cosmetics with HQ medication only available upon prescription^7^. However, concrete evidence supporting these side effects is still lacking^7, 39^.

The US FDA lists some HQ-containing treatments as OTC drugs^3^. HQ remains one of the most frequently used and most effective skin-lightening products to treat skin hyperpigmentation disorders^5^. Topical HQ application is often accompanied with local skin irritation, which causes discomfort and hinders its usage among certain patients^10^. A recent double-blind randomized comparative study showed that skin irritation occurred in over 30% of patients who received topical HQ treatment for PIH^8^. Topical steroids are oftentimes co-applied with HQ to help reduce its irritancy^2, 3^. However, long term usage of topical steroid is associated with side effects, including skin atrophy^11, 40^. Our study revealed for the first time that HQ can cause irritation or pain through TRPA1 activation. Furthermore, pharmacological blockage of TRPA1 almost completely abolished HQ-induced nocifensive behavior in mice. Therefore, our findings suggest that blocking TRPA1 by topical application of TRPA1 specific inhibitors may offer a novel and efficient method to reduce the irritation caused by topical HQ treatment.

In summary, our study identifies TRPA1 as a molecular target for the widely used skin-lightening reagent HQ and provides novel insights into HQ-evoked skin irritation. Our study also suggests that topical application of selective TRPA1 antagonist might be potentially used to counteract HQ-induced skin irritation.

## Methods

### Animals

Experimental procedures were approved by the Institutional Animal Care and Use Committees of Duke University. Mice were housed at facilities accredited by the Association for Assessment and Accreditation of Laboratory Animal Care (USA) in standard environmental conditions (12-hour light–dark cycle and 23 °C). Food and water were provided ad libitum. Male C57BL/6 mice (6–8 weeks old) were purchased from Jackson Laboratory (Bar Harbor, ME) and used in the present study. Procedures were approved by the Institutional Animal Care and Use Committee of Duke University, protocol A052-17-02, approved on 03/17/2017. All methods were performed in accordance with the relevant guidelines and regulations of Duke University and Zhejiang Chinese Medical University.

### Chemicals

HQ, ionomycin, capsaicin and mustard oil were obtained from Sigma-Aldrich (St. Louis, MO, USA). HC-030031 and AMG-9810 were obtained from Tocris (Minneapolis, MN, USA).

### Cell culture

Human embryonic kidney (HEK) 293 cells (ATCC, CRL-1573) were cultured in Dulbecco’s modified Eagle’s medium (DMEM, Lonza, Belgium) supplemented with 10% fetal bovine serum (Lonza, Belgium), 2 mM L-glutamine, 100 units/mL penicillin, and 100 μg/mL streptomycin. Cells were transfected by Lipofectamine 2000 (Invitrogen, Carlsbad, CA, USA).

Adult mouse dorsal root ganglia (DRGs) were dissociated using 0.28 Wünsch units/ml Liberase Blendzyme 1 (Roche Diagnostics, Mannheim, Germany), as described previously^41^. Neurons were cultured in Neurobasal-A medium (Invitrogen, Grand Island, NY) with B-27 supplement, 0.5 mmol/L glutamine, and 50 ng/mL nerve growth factor (Calbiochem, La Jolla, CA) on an 8-well chambered coverglass coated with poly-D-lysine (Sigma, St. Louis, MO) and mouse laminin (Invitrogen, Carlsbad, CA, USA).

### Ca^2+^ imaging

For Ca^2+^ imaging of HEK293 cells, cells were used within 48 h after transfection. For DRG neurons, neurons were used 24 h after dissociation. Cells were loaded with Fura 2-AM (10 μM, Invitrogen) for 45 min in a loading buffer containing: NaCl 140, KCl 5, CaCl2 2, MgCl2 2, HEPES 10 (pH 7.4 adjusted with NaOH). Cells were subsequently washed three times and imaged in the loading buffer. Ratiometric Ca2 + -imaging was performed on an Olympus IX51 microscope with a Polychrome V monochromator (Till Photonics) and a PCO Cooke Sensicam QE CCD camera and Imaging Workbench 6 imaging software (Indec). Fura-2 emission images were obtained with exposures of 0.5 ms at 340 nm and 0.3 ms at 380 nm excitation wavelengths. Ratiometric images were generated using ImageJ software. A cell was considered responsive if the peak Ca^2+^ response is above 20% of the baseline.

### Electrophysiological recording

Details of patch clamp recording have been described previously^22^. Briefly, recordings from HEK293T cells were performed with borosilicate glass pipettes with an initial series resistance of 2–4 MΩ after loading the pipette solution. Currents were filtered at 2.3 kHz and digitized at 100 μs intervals using an EPC-10 amplifier and PatchMaster acquisition software (HEKA, Germany). Perforated whole-cell hTRPA1 currents in HEK293 cells were recorded by patch-clamp recordings with a pipette solution containing (in mM): CsAsp 140, MgCl2 2, HEPES 10, EGTA 10, pH 7.4 (CsOH) with ~30 pM amphotericin B added. The perfusion solution (in mM) contained: NaCl 140, KCl 4, EGTA 2, MgCl2 2, HEPES 10, and glucose 8, pH 7.4 (NaOH).

### Nocifensive behavioral assay

Mice were placed into transparent chambers and habituated for 30 minutes before testing. HQ (1–30 mM, 20 µl/paw, dissolved in PBS) was injected into the hind paw of mice using 1-mL syringe and 30-gauge needle in a volume of 20 µl. Acute nocifensive behavior (licking, flinching, or biting of injected paw) was recorded with a video camera for 10 minutes and quantified thereafter.

Mechanical hyperalgesia was examined by von Frey hair analysis. Mice were habituated for 30 minutes to the wire mesh surface before testing. Paw withdrawal thresholds were determined using a series of von Frey filaments (0.008–6.00 g) pressed against the plantar surface of the hind paw in ascending order beginning with the finest fiber following standard procedures^17, 42–44^. The minimum force (g) that caused the mouse to withdraw its hind paw away from the filament was considered as the withdrawal threshold. For each paw, a von Frey hair was applied 5 times at 10-second intervals. The threshold was determined when paw withdrawal was observed in more than 3 of 5 applications. A withdrawal response was considered valid only if the hind paw was removed completely from the platform. If the paw withdrawal response was ambiguous, the application was repeated. All behavioral tests were performed by an experimenter blinded to experimental conditions.

### Statistics

Student’s *t*-test was used for comparison of data between 2 groups. One-way or two-way ANOVA followed by Tukey post hoc test was used for comparison of ≥3 groups. Comparison is considered significantly different if the *p* value is less than 0.05. Data in bar graphs are expressed as means ± S.D.

## References

[1] Plensdorf S, Martinez J (2009). Common pigmentation disorders. Am Fam Physician.

[2] Davis EC, Callender VD (2010). Postinflammatory hyperpigmentation: a review of the epidemiology, clinical features, and treatment options in skin of color. J Clin Aesthet Dermatol.

[3] Chandra M, Levitt J, Pensabene CA (2012). Hydroquinone therapy for post-inflammatory hyperpigmentation secondary to acne: not just prescribable by dermatologists. Acta Derm Venereol.

[4] Draelos ZD (2007). Skin lightening preparations and the hydroquinone controversy. Dermatol Ther.

[5] Sofen B, Prado G, Emer J (2016). Melasma and Post Inflammatory Hyperpigmentation: Management Update and Expert Opinion. Skin Therapy Lett.

[6] Lartey M (2017). Use of skin-lightening products among selected urban communities in Accra, Ghana. Int J Dermatol.

[7] Tse TW (2010). Hydroquinone for skin lightening: safety profile, duration of use and when should we stop?. J Dermatolog Treat.

[8] Yousefi A (2014). Is topical zinc effective in the treatment of melasma? A double-blind randomized comparative study. Dermatol Surg.

[9] Chan R (2008). A randomized controlled trial of the efficacy and safety of a fixed triple combination (fluocinolone acetonide 0.01%, hydroquinone 4%, tretinoin 0.05%) compared with hydroquinone 4% cream in Asian patients with moderate to severe melasma. Br J Dermatol.

[10] Smiles KA (2007). A hydroquinone formulation with increased stability and decreased potential for irritation. J Cosmet Dermatol.

[11] Barnes L, Kaya G, Rollason V (2015). Topical corticosteroid-induced skin atrophy: a comprehensive review. Drug Saf.

[12] Patrick E, Juberg DR, O’Donoghue J, Maibach HI (1999). Depigmentation with tert-butyl hydroquinone using black guinea pigs. Food Chem Toxicol.

[13] Bautista DM, Pellegrino M, Tsunozaki M (2013). TRPA1: A gatekeeper for inflammation. Annu Rev Physiol.

[14] Liu B (2013). TRPA1 controls inflammation and pruritogen responses in allergic contact dermatitis. FASEB J.

[15] Bessac BF (2008). TRPA1 is a major oxidant sensor in murine airway sensory neurons. J Clin Invest.

[16] Bessac BF (2009). Transient receptor potential ankyrin 1 antagonists block the noxious effects of toxic industrial isocyanates and tear gases. FASEB J.

[17] Liu B (2016). Oxidized Phospholipid OxPAPC Activates TRPA1 and Contributes to Chronic Inflammatory Pain in Mice. PLoS One.

[18] Kanju P (2016). Small molecule dual-inhibitors of TRPV4 and TRPA1 for attenuation of inflammation and pain. Sci Rep.

[19] Kistner K (2016). Systemic desensitization through TRPA1 channels by capsazepine and mustard oil - a novel strategy against inflammation and pain. Sci Rep.

[20] Miura S (2013). Involvement of TRPA1 activation in acute pain induced by cadmium in mice. Mol Pain.

[21] Bautista DM (2006). TRPA1 mediates the inflammatory actions of environmental irritants and proalgesic agents. Cell.

[22] Gui J (2014). A tarantula-venom peptide antagonizes the TRPA1 nociceptor ion channel by binding to the S1-S4 gating domain. Curr Biol.

[23] Xu H, Delling M, Jun JC, Clapham DE (2006). Oregano, thyme and clove-derived flavors and skin sensitizers activate specific TRP channels. Nat Neurosci.

[24] Gavva NR (2005). AMG 9810 [(E)-3-(4-t-butylphenyl)-N-(2,3-dihydrobenzo[b][1,4] dioxin-6-yl)acrylamide], a novel vanilloid receptor 1 (TRPV1) antagonist with antihyperalgesic properties. J Pharmacol Exp Ther.

[25] Liu B (2016). IL-33/ST2 signaling excites sensory neurons and mediates itch response in a mouse model of poison ivy contact allergy. Proc Natl Acad Sci USA.

[26] Matsubayashi T (2003). Intradermal concentration of hydroquinone after application of hydroquinone ointments is higher than its cytotoxic concentration. Biol Pharm Bull.

[27] Atoyan R, Shander D, Botchkareva NV (2009). Non-neuronal expression of transient receptor potential type A1 (TRPA1) in human skin. J Invest Dermatol.

[28] Hox V (2013). Crucial role of transient receptor potential ankyrin 1 and mast cells in induction of nonallergic airway hyperreactivity in mice. Am J Respir Crit Care Med.

[29] Caceres AI (2009). A sensory neuronal ion channel essential for airway inflammation and hyperreactivity in asthma. Proc Natl Acad Sci USA.

[30] Story GM (2003). ANKTM1, a TRP-like channel expressed in nociceptive neurons, is activated by cold temperatures. Cell.

[31] Plonka PM, Handjiski B, Michalczyk D, Popik M, Paus R (2006). Oral zinc sulphate causes murine hair hypopigmentation and is a potent inhibitor of eumelanogenesis *in vivo*. Br J Dermatol.

[32] Plonka PM, Handjiski B, Popik M, Michalczyk D, Paus R (2005). Zinc as an ambivalent but potent modulator of murine hair growth *in vivo*- preliminary observations. Exp Dermatol.

[33] Hu H, Bandell M, Petrus MJ, Zhu MX, Patapoutian A (2009). Zinc activates damage-sensing TRPA1 ion channels. Nat Chem Biol.

[34] Zhou J, Feng JY, Wang Q, Shang J (2015). Calcitonin gene-related peptide cooperates with substance P to inhibit melanogenesis and induces apoptosis of B16F10 cells. Cytokine.

[35] Ping F, Shang J, Zhou J, Song J, Zhang L (2012). Activation of neurokinin-1 receptor by substance P inhibits melanogenesis in B16-F10 melanoma cells. Int J Biochem Cell Biol.

[36] Smith CJ, O’Hare KB, Allen JC (1988). Selective cytotoxicity of hydroquinone for melanocyte-derived cells is mediated by tyrosinase activity but independent of melanin content. Pigment Cell Res.

[37] Land EJ, Ramsden CA, Riley PA (2004). Quinone chemistry and melanogenesis. Methods Enzymol.

[38] Slominski A, Tobin DJ, Shibahara S, Wortsman J (2004). Melanin pigmentation in mammalian skin and its hormonal regulation. Physiol Rev.

[39] O’Donoghue JL (2006). Hydroquinone and its analogues in dermatology - a risk-benefit viewpoint. J Cosmet Dermatol.

[40] Mehta AB (2016). Topical corticosteroids in dermatology. Indian J Dermatol Venereol Leprol.

[41] Willis DN, Liu B, Ha MA, Jordt SE, Morris JB (2011). Menthol attenuates respiratory irritation responses to multiple cigarette smoke irritants. FASEB J.

[42] Pitcher GM, Ritchie J, Henry JL (1999). Paw withdrawal threshold in the von Frey hair test is influenced by the surface on which the rat stands. J Neurosci Methods.

[43] Liu B (2013). TRPM8 is the principal mediator of menthol-induced analgesia of acute and inflammatory pain. Pain.

[44] Caceres AI (2017). Transient Receptor Potential Cation Channel Subfamily M Member 8 channels mediate the anti-inflammatory effects of eucalyptol. Br J Pharmacol.

